# Understanding the ionic activity and conductivity value differences between random copolymer electrolytes and block copolymer electrolytes of the same chemistry†

**DOI:** 10.1039/d1ra02519h

**Published:** 2021-04-22

**Authors:** Mario V. Ramos-Garcés, Ke Li, Qi Lei, Deepra Bhattacharya, Subarna Kole, Qingteng Zhang, Joseph Strzalka, Polyxeni P. Angelopoulou, Georgios Sakellariou, Revati Kumar, Christopher G. Arges

**Affiliations:** Cain Department of Chemical Engineering, Louisiana State University Baton Rouge LA 70803 USA carges@lsu.edu; Department of Chemistry, Louisiana State University Baton Rouge LA 70803 USA revatik@lsu.edu; X-ray Science Division, Argonne National Laboratory Lemont IL 60439 USA; Department of Chemistry, National and Kapodistrian University of Athens 15771 Athens Greece

## Abstract

Herein, a systematic study where the macromolecular architectures of poly(styrene-*block*-2-vinyl pyridine) block copolymer electrolytes (BCE) are varied and their activity coefficients and ionic conductivities are compared and rationalized *versus* a random copolymer electrolyte (RCE) of the same repeat unit chemistry. By performing quartz crystal microbalance, ion-sorption, and ionic conductivity measurements of the thin film copolymer electrolytes, it is found that the RCE has higher ionic activity coefficients. This observation is ascribed to the fact that the ionic groups in the RCE are more spaced out, reducing the overall chain charge density. However, the ionic conductivity of the BCE is 50% higher and 17% higher after the conductivity is normalized by their ion exchange capacity values on a volumetric basis. This is attributed to the presence of percolated pathways in the BCE. To complement the experimental findings, molecular dynamics (MD) simulations showed that the BCE has larger water cluster sizes, rotational dynamics, and diffusion coefficients, which are contributing factors to the higher ionic conductivity of the BCE variant. The findings herein motivate the design of new polymer electrolyte chemistries that exploit the advantages of both RCEs and BCEs.

## Abstract 2

Random copolymer electrolytes have better permselectivity but lower ionic conductivity than block copolymer electrolytes of the same repeat unit chemistry.

## Graphical abstract

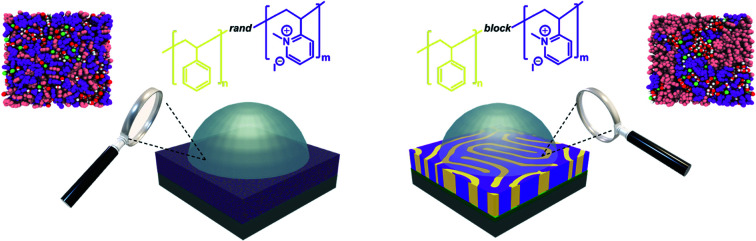

## Introduction

Macromolecular architectures of polymer electrolytes used in ion-exchange membranes (IEMs) have a profound impact on ionic conductivity^1^ and other transport properties such as permselectivity and osmotic drag.^2,3^ Ionic conductivity dictates ohmic resistances in electrochemical separations and thus contributes to the overall energy efficiency of these units.^4,5^ Permselectivity, on the other hand, influences the current utilization in electrochemical separation units.^3^ It is worth noting that anion exchange membranes (AEMs) and cation exchange membranes (CEMs) exposed to low concentration aqueous salt solutions are permselective (>0.9) for anions and cations respectively.^4^ However, there is significant interest in designing new IEMs for electrochemical separations that discriminate ions based on chemistry when they have the same valence^6^ (*e.g.*, Li^+^*versus* Na^+^).^7^ On top of these transport considerations, IEMs require mechanical integrity,^3,8^ in the presence of liquids of varying composition (*e.g.*, water–organic mixtures) and total dissolved salt (TDS) concentrations. These membranes often physically separate two liquid compartments in electrodialysis^3^ and electrodeionization^9,10^ or electrodes when used in membrane capacitive deionization.^4^

A subset of polymeric materials that has received significant attention includes block copolymer electrolytes (BCEs)^11,12^ as their percolated pathways of ionic domains ameliorate ionic conductivity and the non-ionic domains foster mechanical properties and curtail excess swelling. Although several studies^1,11,13,14^ exist comparing the ionic conductivity of random/amorphous polymer electrolytes (RCEs) and microphase separated BCEs (as well as aligned and anti-aligned ionic domains),^12^ there is a lack of studies dedicated to the ionic activity differences within these materials with systematically varied macromolecular architectures – especially when the repeat unit chemistries are the same. Ionic activity is particularly important because this thermodynamic property strongly influences selectivity and ionic transport properties.^15–18^ Notably, there have been numerous studies using Manning's theory of counterion condensation^19–26^ (or variations of this theory)^27–29^ to predict or determine the activity coefficients of ions in IEMs. However, Manning's theory does not always yield accurate values (*e.g.*, perfluorosulfonic acid membranes exposed to concentrated acid solutions).^30^

In our previous work,^31^ we reported a multitude of techniques, such as a quartz crystal microbalance (QCM), environmental grazing incidence small-angle X-ray scattering (GI-SAXS) and molecular dynamics (MD) simulations, for probing ionic activity in a model thin film block copolymer electrolyte composed of poly(styrene-*block*-2 vinyl pyridine-*co-n*-methyl pyridinium iodide) (PS*b*P2VP/NMP^+^I^−^). The activity coefficients of this thin film BCE in the osmotic-controlled regime^32^ were found to be low, but 90% of the ions did exert activity in this regime (*i.e.*, a small contingent of condensed counterions that do not exert activity). The low activity coefficients were rationalized by the lack of water uptake and solvation to further dissociate the ion charge pairs. These properties are vital for mediating ion transport in the ion-containing polymer.^33^ It is important to note that thin film studies were performed because they mimic the same structures in bulk polymeric ion-exchange membrane separators^34^ and are amenable for producing materials with long-range order and aligned ionic domains.^13,35^ Herein, we study the differences in ionic activity of self-assembled BCE thin films of PS*b*P2VP/NMP^+^I^−^ with different domain sizes *versus* RCE thin films of the same chemistry. By using QCM, GI-SAXS, io-sorption experiments, MD simulations, and conductivity measurements, we show that the RCE has higher ionic activity coefficients while the BCE demonstrate higher ionic conductivity. The difference in conductivity is ascribed to the lack of percolated pathways in the RCE while its higher activity coefficients is rationalized by its overall lower charge density of the chain caused by the larger distance between ionic moieties along the polymer backbone (on average).

## Materials and methods

### Materials

Poly(styrene-*random*-2-vinyl pyridine) with *M*_n_ value of 130k and a PDI of 1.69 was acquired from Sigma-Aldrich. Poly(styrene-*block*-2-vinyl pyridine) (PS*b*P2VP) and poly(2-vinyl pyridine-*block*-styrene-*block*-2-vinyl pyridine) (P2VP*b*PS*b*P2VP) with *M*_n_ values of 102–97k (PDI: 1.12), 40–44k (PDI: 1.10) and 12–23–12k (PDI: 1.25), respectively, were purchased from Polymer Source Inc. and were used as is. Silicon wafers with and without thermally grown oxide layers of 1 μm were acquired from WRS Materials. The silicon wafers used for atomic force microscopy, ion sorption, and GI-SAXS were p-type and doped with boron (resistivity of 1 to 20 Ω cm). The silicon wafers with 1 μm SiO_*x*_ were used for fabricating interdigitated electrodes (IDEs). Gold quartz crystals (5 MHz) were supplied from Gamry. 99.9% gold pellets were purchased from ACI Alloys for making IDEs. Iodomethane (CH_3_I), potassium iodide (KI), toluene, *N*,*N*-dimethylformamide (DMF) were acquired from VWR or Sigma-Aldrich and used as is. S1813 photoresist and MF-319 developer were purchased from MicroChemicals.

### Procedures

The procedure to prepare interdigitated electrodes (IDEs), BCE thin films, as well as methods for thin film characterization (*e.g.*, solution uptake/water *via* QCM, ion sorption, environmental GI-SAXS, and ionic conductivity using IDE substrates) are documented in our previous report.^31^ More specifically, solution uptake (SU) experiments were performed using a Gamry e-chem quartz crystal microbalance (QCM) where the BCE and RCE thin films were prepared on gold quartz crystals. These crystals were then loaded into the QCM chamber and a KI_aq_ solution was added so that the thin films were submerged in the solution. The frequency shift of the polymer was monitored and converted into mass gain. Then, this mass gain was used to determine the swelling uptake. All experiments were performed at room temperature. The equations and methods for calculations are provided in our previous report.^31^

GI-SAXS experiments were performed at the Advanced Photon Source (APS) Beamline 8-ID-E at Argonne National Laboratory. The beamline was equipped with an environmental chamber and the BCE thin films self-assembled on Si wafers, were covered with KI_aq_ droplets. The grazing incidence angle was 0.11°. Our previous work provides details on data analysis and equations used.^31^ Ion sorption experiments were performed to determine the counterion (I^−^) and co-ion (K^+^) concentrations in the thin films. For this, the BCE thin films were self-assembled or the RCE thin film prepared on 1 inch diameter Si wafers. Afterwards, the wafers were loaded into plexiglass cells that can be sealed and had an injection port.^31^ Then, 0.5 mL of KI_aq_ was placed on the surface of the wafer and after 24 hours, the KI_aq_ solution was removed with a syringe. 1 mL of DI water was then injected into the chamber and placed on top of the wafers. The DI water was interfaced with the BCE and RCE thin films for 24 hours and syringed out for analysis. The K^+^ concentration was determined with inductively coupled plasma-optical spectroscopy (ICP-OES) by using the signal from potassium. The counterion concentration (I^−^) were determined by dissolving the thin films with 5 mL of DMF after they were exposed to KI_aq_ and remove the solution. For this, the chambers containing DMF on top of the Si wafers were sonicated for 10 min to dissolve all the polymer into the solvent. The I^−^ concentration was measured using liquid chromatography-mass spectrometry (LCMS). Electron micrographs were collected on the BCEs samples with a FEI Quanta SEM/FIB microscope at 5 kV and a working distance of 5 mm using a scattered electron detector.

Ionic conductivity measurements for the RCE and BCEs were performed on IDEs substrates. For this, the samples were prepared onto the IDEs in the exact same manner as in Si wafers. The IDEs with the thin films were placed in a sealed, home-built stainless-steel chamber that had temperature and humidity control.^31^ The chamber also had a temperature probe and electrical contacts for making ionic conductivity measurements for the thin films on IDEs. 100% relative humidity (RH) with a nitrogen carrier gas was delivered to the testing chamber at 1 L min^−1^ through control of the dew point temperature on the bubbler. Measurements were performed at 27 °C. The thin film resistance for each thin film was determined using electrochemical impedance spectroscopy (EIS) carried out in galvanostatic mode. The electrode pad areas of the IDE substrate were scraped away with a cotton Q-tip to remove the film for electrical connections and the conductivity was calculated using the following formula:^31^

1 *σ* = *d*/(*n* – 1) × *R*_flim_ × *l* × *n*_film_

where, *d* = distance between teeth on IDEs, *n* = number of teeth on IDEs, *R*_film_ = resistance of BCE and RCE thin films, *l* = length of the teeth on IDEs, *n*_film_ = thickness of BCE and RCE thin films

Note: The thickness of the BCE and RCE samples were determined with ellipsometry on Si wafers.

### Sample preparation

Mono-hydroxy terminated PS*r*P2VP (OH–PS*r*P2VP) were synthesized *via* nitroxide mediated polymerization (NMP).^13,36^ Thin film RCEs were prepared in a similar fashion to thin film BCEs described in our previous work.^31^ More specifically, a 1.5 wt% solution of PS*r*P2VP in toluene was prepared and spincoated (4000 rpm for 45 seconds) onto a Si wafer, gold quartz crystals or IDEs. After spin coating the RCP on the substrate, the sample was exposed to 200 °C for 10 minutes under a dry nitrogen gas. After grafting the PS*r*P2VP brushes to the substrate, PS*b*P2VP 40–44k, PS*b*P2VP 102–97k or P2VP*b*PS*b*P2VP 12–23–12k was spincoated on the substrates and then solvent annealed with acetone to make perpendicular lamellae structures.^31^ Afterwards, the thin film random copolymer and block copolymer samples were exposed to CH_3_I vapor for 24 hours in a 125 mL wide mouth jar. This exposure alkylated the nitrogen in the pyridine ring of the polymers to prepared *n*-methyl pyridinium iodide groups (*i.e.*, fixed charge groups) without disrupting the nanostructure of the BCPs and forming RCE and BCE samples.

### Molecular dynamics simulations

#### Simulation method

In order to explore the molecular origins of the differences in the behavior between the RCE and BCE systems, all atom molecular dynamics (MD) simulations as well as replica exchange^37^ molecular dynamics (REMD) simulations were carried out on representative BCE and RCE systems (*vide infra*). The BCE case consisted of polymer chains each with a block of 20 hydrophobic styrene units followed by 20 hydrophilic units of alternating uncharged pyridine and charged pyridinium (with iodide as the counterion) moieties. The RCE case had the same number of hydrophobic and hydrophilic moieties but consisted of a repeating unit of styrene–pyridine–styrene–pyridinium. While these chains (see Fig. S2a† for the simulation RCE and BCE schematic) are much shorter than the experimental systems and the RCE is not a truly random chain, they retain the essential features/motifs of the experimental system, namely a clear hydrophilic and hydrophobic region for the BCE and a mixed case for the RCE case. For each of the two cases, the simulation box consisted of 30 polymer chains, an iodide counterion for each tethered positively charged pyridinium moiety and 6 waters per pyridinium group. The water amount was based on the solution uptake experiments. The systems were simulated using the OPLSAA^38^ force-field along with the TIP3P^39^ model for water using the LAMMPS^40^ software code. From the replica exchange simulation trajectories at 300 K the solvation structure as well as counterion condensation was calculated. The rotational and translational dynamics of the system were studies using the trajectories from the canonical MD simulations at constant temperature. Finally, non-equilibrium simulations in the presence of an electric field were carried out to determine the ionic conductivity values.^31,41^

#### Simulation details

The initial structure of the BCE and RCE polymer chains were generated by the Avogadro software,^42^ and then optimized in the NVT (300 K) ensemble for 100 ps. With the help of the Packmol software,^43^ the optimized BCE and RCE chains with the iodide counterion and water molecules were packed into a cubic simulation box with the box length around 100 Å. The SHAKE^44^ algorithm was used to constrain the bond lengths and bond angles for the water molecules. The LAMMPS^40^ software was used to carry out the simulations. Equilibration was carried out by first simulating each system in the NVT ensemble for 5 ns at a temperature of 300 K using the Nosé–Hoover thermostat followed by a 30 ns simulation in the NPT ensemble (temperature of 300 K and pressure of 1 atm) *via* the Nosé–Hoover thermostat and barostat.^45,46^ These were then used as initial configurations for the replica exchange^31^ simulations that were then carried out with 16 replica systems equally distributed in temperature between 290 K to 365 K for 20 ns for each temperature for better sampling for structural data. In addition, 30 ns production runs in the NVT ensemble were carried out to obtain the dynamical properties of the systems under study. Finally, to get the conductivity of the BCE and RCE, non-equilibrium MD simulations were performed by adding an electric field of 0.1 V Å^−1^ in the *z* direction for 20 ns. The electrostatic interactions were calculated using the Ewald method, specifically PPPM.^47^

#### Electronic structure calculations

The partial charges on the polymers were determined by fitting to the *ab initio* electrostatic potential on a grid around the polymer electrolyte unit molecule (a single repeating unit of the polymer), using the CHELPG^48^ scheme at the HF/6-31G* level with the GAUSSIAN 09 (ref. 49) software. The reason for choosing this scheme is to maintain consistency with the OPLSAA force field.

## Results and discussion

**Fig. 1:**
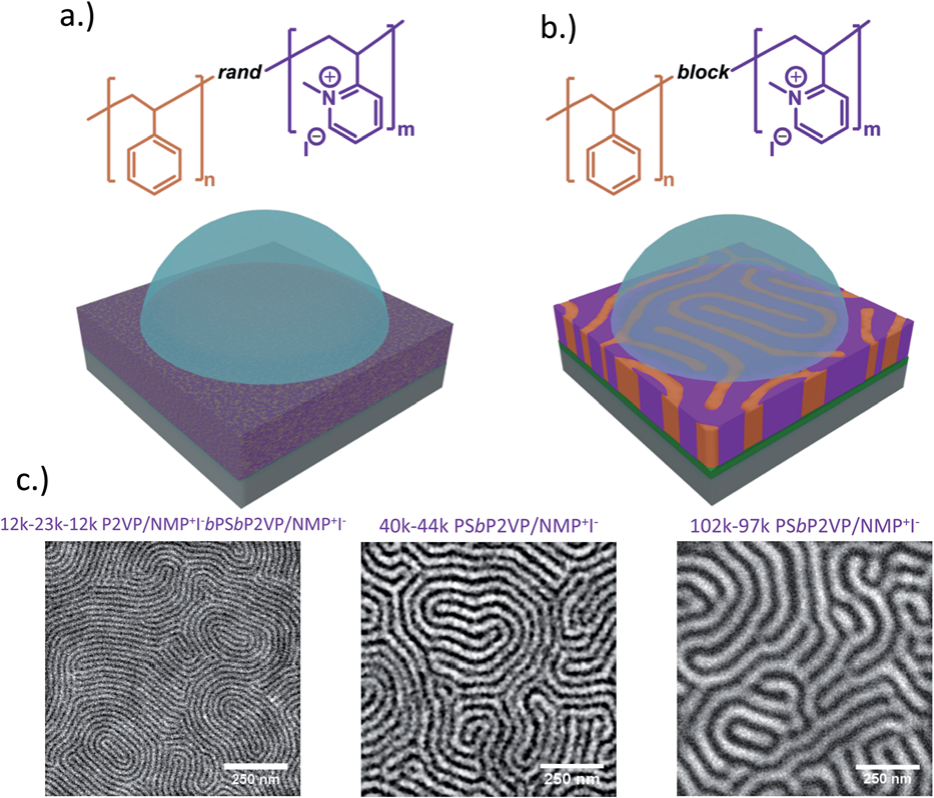
(a) Depiction of RCE and (b) BCE thin films interfaced with KI_aq_ solution. (c) Electron micrographs of BCEs with different *M*_n_ values and period feature sizes.

The molecular architectures of RCEs and BCEs thin films on substrate surfaces interfaced with KI_aq_ droplets are depicted in Fig. 1a and b. Unlike the RCE, the BCE is microphase separated with the ionic moieties aggregated into periodic domains that vary in size depending on the degree of polymerization of the BCE.^50^ Since the block copolymer starting materials had *M*_n_ values that were roughly equal for each constituent (styrene and 2-vinyl pyridine), the BCEs formed perpendicular lamellae on the non-preferential layers (Fig. 1c). The periodic spacing (*L*_0_) for the 12–23–12k P2VP/NMP^+^I^−^*b*PS*b*P2VP/NMP^+^I^−^, 40–44k PS*b*P2VP/NMP^+^I^−^ and 102–97k PSbP2VP/NMP^+^I^−^ BCEs under vacuum were 21.3 nm, 44.0 nm, and 69.0 nm, respectively, and were determined by the Fast Fourier Transform (FFT) of the electron micrographs.

**Fig. 2:**
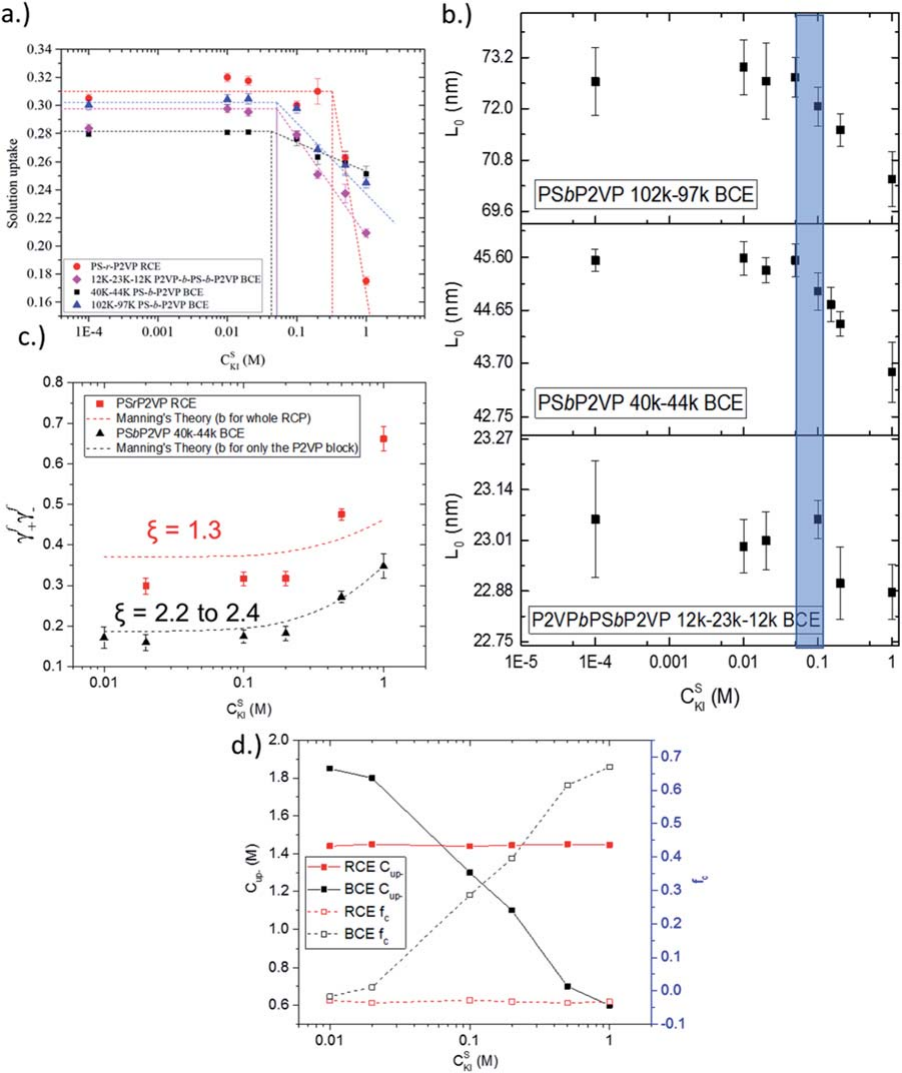
(a) Solution uptake of RCE and BCE chains as a function of external KI_aq_ concentration. (b) *L*_0_ (from GI-SAXS) *versus* external KI_aq_ concentration for all BCE samples. (c) Measured activity coefficients of counterions and co-ions in the RCE and PS*b*P2VP 40–44k BCE thin films. The Manning parameter (*ξ*) is shown for the RCE and BCE. (d) The concentration of uncondensed counterions (*C*_up-_) along the polymer chain in the RCE and PS*b*P2VP 40–44k BCE films as a function of external KI_aq_ concentration determined from the Gibbs–Donnan equilibrium expression (left axis) and the fraction of uncondensed counterions (*f*_u_) (right axis).

The solution uptake value of the RCE film and BCE films of different periodicity are given in Fig. 2a. Notably, the KI_aq_ concentration needed to transition the RCE from the osmotic-controlled regime to the condensed-controlled regime was over 3× larger than for the BCE samples (0.20 M *versus* ∼0.06 M). The BCE samples experienced the transition between the two regimes at about the same external KI_aq_ concentration (0.05 M to 0.065 M). A similar observation was observed for the BCEs from environmental GI-SAXS experiments (Fig. 2b), but the transition between the two regimes had a higher upper bound and a larger range (0.05 M to 0.095 M, shaded in blue in Fig. 2b). The environmental GI-SAXS experiments identified the external solution concentration on the BCE samples that caused the BCE to undergo deswelling (*i.e.*, the transition point) and thus shrinkage of the BCEs' periodic domain spacing value (*L*_0_).^15,31^ The two key takeaways from Fig. 2a and b are (i) the ion activity is higher in RCEs because a larger external solution concentration is needed to overcome the osmotic pressure in the film (which causes film deswelling) and (ii) the ion activity in BCEs is not a function of the BCE *L*_0_ value. Due to the observations in Fig. 2a and b, only the RCE *versus* the 40–44k BCE will be compared since the differences in the activity of the BCEs, inferred from swelling behaviour, with different BCE periods were not different.

The actual activity coefficients were determined directly using ion-sorption experiments as described in our previous work.^31^ This approach directly measures the concentration of ions in the thin film polymer samples (see Fig. S1†) and uses the known activity values of ions in the external solution.^31^Fig. 2c shows that the activity coefficients of ions in the RCE sample were ∼1.8 times larger than the 40–44k PS*b*P2VP/NMP^+^I^−^ BCE sample. The predicted activity coefficient values from Manning's theory of counterion condensation are also presented in Fig. 2c. Notably, Manning's theory was more accurate for predicting the measured activity coefficient values of BCEs when compared to the RCEs. The Manning parameter (*ξ*) was also determined for the RCE and BCE materials. *ξ* is a dimensionless value that normalizes the Bjerrum length (*λ*_b_) to the average distance between fixed charges on the polymer backbone (*b*). By using the formula *ξ* = *λ*_B_/*b* the Manning parameter was determined and found that the BCE has a Manning parameter that is 2 times that of the RCE. This difference is accounted for by the larger *b* value for the RCE over the BCE. The Manning parameter values can be found on Fig. 2c. It is worth mentioning that values higher than 1 (the critical value for monovalent salts) indicate a reduction in activity coefficients. The smaller *ξ* parameter for the RCE accounts for its higher ionic activity coefficients over the BCE. Additionally, the Gibbs–Donnan model was used to determine the concentration (*C*_up-_) and fraction (*f*_u_) of *n*-methyl pyridinium groups in the polymer that were dissociated (Fig. 2d). This model utilized the directly measured activity coefficient values given in Fig. 2c. From Fig. 2d, it can be seen that the RCE sample had 100% of its ionic groups dissociated and these dissociated ionic groups exerted activity across the concentration range of KI_aq_ interfaced with the RCE. The BCE sample, on the other hand, has 90% of its ionic groups dissociated in the osmotic regime (*i.e.*, KI_aq_ external is less than 0.05 M). It experiences a further reduction in the *C*_up-_ and *f*_u_ upon transitioning to the condensation regime (*i.e.*, increasing the concentration of ions in the external solution >0.05 M). The large activity values of *n*-methyl pyridinium iodide in the RCE sample *versus* the BCE sample in Fig. 2c account for the 3× larger KI_aq_ transition point from the osmotic-regime to the condensation regime in Fig. 2a and b and the greater *C*_up-_ and *f*_u_ observed in Fig. 2d.

**Fig. 3:**
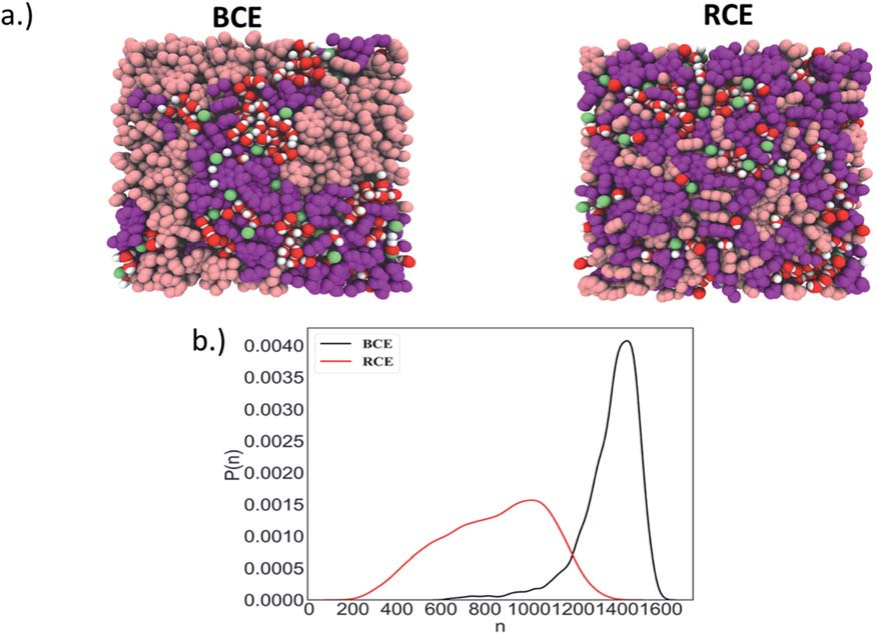
(a) The model BCE (left) and RCE (right) with the different chemical groups (pink: styrene repeat units, purple: pyridine/*n*-methyl-pyridinium iodide repeat units, green: iodide counterion, red-white: water). (b) Probability distribution, *P*(*n*), of the largest water clusters as a function of the number of water molecules (*n*) in the clusters of BCE and RCE.

As stated above, Manning's theory of counterion condensation predicted the activity coefficients of BCE samples with greater accuracy over the RCE sample. We rationalize this observation based on the fact that Manning's theory was developed for idealized polymer electrolytes in solutions for predicting colligative properties.^51^ We assert that the microphase separated ionic domains in BCEs mimic concentrated polymer electrolyte solutions; and thus, account for the better agreement between theory and experimental observation. The ionic groups in RCEs are more spaced on average and a representative snapshot from molecular dynamics (MD) simulations (see Fig. S2a† for the model RCE and BCE chains used in these simulations), presented in Fig. 3a, show more distributed and discrete accumulation of water. Hence, the RCE has less of a resemblance of polymer electrolytes in solution and thus Manning's theory is less accurate for predicting the RCE activity coefficient values.

In order to compare the level of hydration around the ions in both the BCE and RCE cases from MD simulations, a robust definition of the hydration shell is needed. The first minimum in the atom (I^−^ or C atom of the methyl group attached to the pyridinium N atom) – water O atom radial distribution function defines the first hydration shell around the I^−^/pyridinium ions (see Fig. S2b†). If the distance is less than this cut-off, the water is considered to be in the first hydration shell of the ions in the BCE and RCE (see ESI† for more details). The average number of waters in the first hydration shell of both the I^−^ and pyridinium cation are tabulated in Table 1. The first hydration shell of I^−^ and C of pyridinium in the BCE case has more water molecules on average compared to the RCE case.

**Table 1:** Comparison of solvation and dynamical properties between BCE and RCE from atomistic simulations

	BCE	RCE
Largest water cluster size	1372 ± 135	842 ± 237
Water diffusion coefficient (Å^2^ ns^−1^)	25.1 ± 0.9	22.9 ± 0.3
Water rotational constant (ps)	87	103
Iodide diffusion coefficient (Å^2^ ns^−1^)	1.12 ± 0.10	1.07 ± 0.08
Iodide conductivity (mS cm^−1^)	26	21
Iodide hopping rate (ns^−1^)	51	51
Iodide hopping rate with electric field (ns^−1^)	131	108
Average number of waters in the first hydration shell around pyridinium	3.52	3.05
Average number of waters in the first hydration shell around I^−^	4.36	4.22

It is important to note that MD simulations do not make a distinction of condensed and uncondensed ions that is often done in experimental studies involving Manning's theory. The MD simulations provides information about the spatial distribution and solvation of counterions as well as the solvation of the fixed ionic groups along the polymer backbone – which is useful for gaining a molecular level understanding of the ionic activity and conductivity values measured in experiments. In the next MD simulation analyses, the distribution and dynamics of water within the BCEs and RCEs were analysed.

The simulation trajectory of the BCE showed distinct water rich interconnected hydrophilic domains and water poor hydrophobic regions (*i.e.*, where the aggregation of styrene takes place), while water in RCE is more clustered as is clear from the representative simulation snapshots in Fig. 3a. To quantify this, the largest water cluster of hydrogen bonded water molecules was calculated for each snapshot for both the BCE and RCE cases. A water molecule is considered to be hydrogen bonded to another if an intermolecular O–H length between them is less than 2.5 Å. The average number of water molecules in the largest cluster for each case is given in Table 1 while the distribution of the size of the largest water clusters is shown in Fig. 3b. The BCE case clearly shows a significantly larger interconnected water network as compared to the RCE case. In addition, the water translational and rotational dynamics are faster for the BCE case as is clear from the values of the water self-diffusion constant (see data in Table 1). A larger value corresponds to faster translation, while smaller water rotational time constants correspond to faster rotational dynamics. The details of the calculations of these dynamical quantities are outlined in the ESI (see Fig. S3 and S4†). From Table 1, it is clear that the iodide diffusion constant is slightly higher in the case of the BCE polymer as compared to the RCE case, whereas the iodide hopping rate in the absence of an applied electric field is the same for both cases. Hopping is considered to take place only if the iodide hops to a new pyridinium ion in its solvation shell (see ESI and Fig. S5† for details) as compared to the previous solvation shell and it does not go back in the next step to the pyridinium ion in the previous solvation shell. In the presence of an electric field, the iodide hopping rate is higher for the BCE case compared to that of the RCE. Combining this data with the knowledge that interconnected water yields higher orientational mobility, suggests that ion migration due to hopping along the chain in the presence of an electric field is mediated by the less restricted water in the BCE case. This faster ion hopping contributes to a higher conductivity for BCE when compared to RCE (see Table 1).

**Fig. 4:**
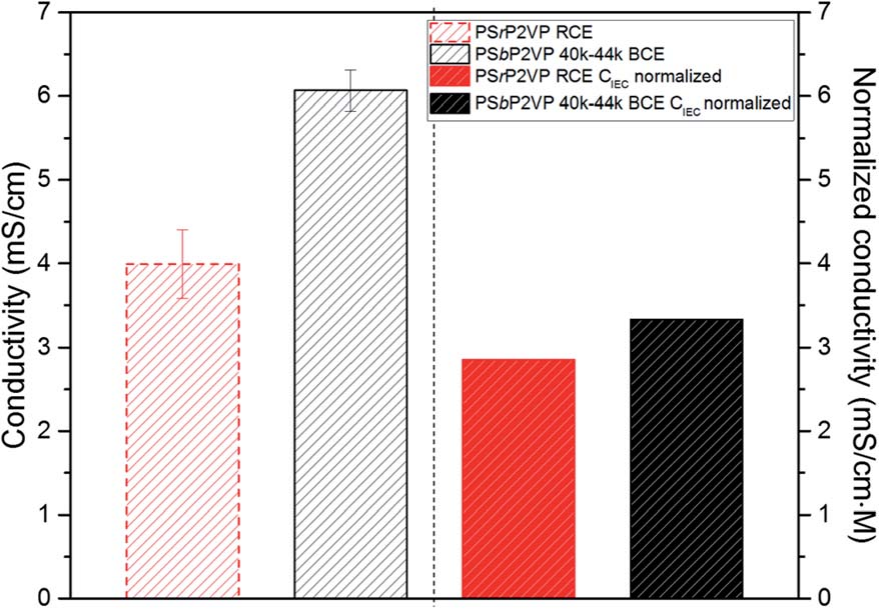
Ionic conductivity (left) and normalized conductivity (right) of RCE and PS*b*P2VP 40–44k BCE thin films on IDEs substrates at 100% RH.

To corroborate the MD simulation findings, ionic conductivity measurements were performed on interdigitated electrodes (IDEs) as described in our previous works.^12,31^ These measurements were performed under 100% relative humidity (RH) at 27 °C. The conductivity values obtained under the specified conditions for the RCE and the PS*b*P2VP 40–44k BCE are provided in Fig. 4. Specifically, the BCE shows 50% higher conductivity (6.1 mS cm^−1^ ± 0.25 mS cm^−1^) over the RCE (4 mS cm^−1^ ± 0.41 mS cm^−1^). We further studied these results by normalizing the ionic conductivity with ion exchange capacities (*C*_IEC_; volumetric basis) of the RCE and BCE. These values were taken from the ion sorption experiments in Fig. S1.† The normalized values shown in Fig. 4 also confirm the higher conductivity of the BCE sample but only at 17% enhancement (3.33 mS cm^−1^ M^−1^*vs.* 2.85 mS cm^−1^ M^−1^). These normalized values are a proxy for the diffusion rate of ions within both the RCE and BCE.^2,31^

## Conclusions

In summary, we show that RCEs display larger ionic activity coefficient values over BCEs with the same repeat unit chemistry. Further, Gibbs–Donnan analysis shows that 100% of ions are exerting activity in RCEs (*i.e.*, no population of condensed counterions). These observations, as well as differences in matching predictions from Manning's theory, are largely dependent upon the distribution of water in the polymer structure. Despite RCEs having better permselectivity, they showed lower ionic conductivity due to lack of percolated pathways of water within the material. Our MD simulations also revealed larger rotational dynamics of water in BCEs – which are another contributing factor to the higher ionic conductivity of this material. Overall, these findings motivate the design of new polymer electrolyte chemistries that exploit the advantages of both RCEs and BCEs – *e.g.*, ionic blocks featuring charge groups that are more spaced out and contain non-ionic moieties that promote water uptake and ionic dissociation.

## Author contributions

M. V. R.-G. and Q. L. performed all experiments, S. K. fabricated IDEs, D. B., J. S., and Q. Z. assisted with GI-SAXS, K. L. and R. K. performed MD simulations, G. S and P. A. synthesized random copolymer brushes, M. V. R.-G. and C. G. A. wrote the manuscript, all authors reviewed and edited the manuscript.

## Conflicts of interest

There are no conflicts to declare.

## Supplementary Material

RA-011-D1RA02519H-s001
