# Supplementary material for: Understanding the ionic activity and conductivity value differences between random copolymer electrolytes and block copolymer electrolytes of the same chemistry
Source: RSC Adv. 2021 Apr 22;11(25):15078–84. doi: 10.1039/d1ra02519h (PMC8697982; doi:10.1039/d1ra02519h)
Supplement: RA-011-D1RA02519H-s001 [file RA-011-D1RA02519H-s001.pdf]

Electronic Supplementary Material (ESI) for RSC Advances.
This journal is © The Royal Society of Chemistry 2021

Electronic Supplementary Information (ESI)

Understanding the ionic activity and conductivity value differences between random copolymer electrolytes and block copolymer electrolytes of the same chemistry

Mario V. Ramos-Garcés[a], Ke Li[b], Qi Lei[a], Deepra Bhattacharya[a], Subarna Kole[a], Qingteng Zhang[c], Joseph Strzalka[c], Polyxeni P. Angelopoulou[d], Georgios Sakellariou[d], Revati Kumar*[b] and Christopher G. Arges*[a]

[a]Cain Department of Chemical Engineering, Louisiana State University, Baton Rouge, LA 70803.
[b]Department of Chemistry, Louisiana State University, Baton Rouge, LA 70803.
[c]X-ray Science Division, Argonne National Laboratory, Lemont, IL 60439.
[d]Department of Chemistry, National and Kapodistrian University of Athens, 15771 Athens, Greece.

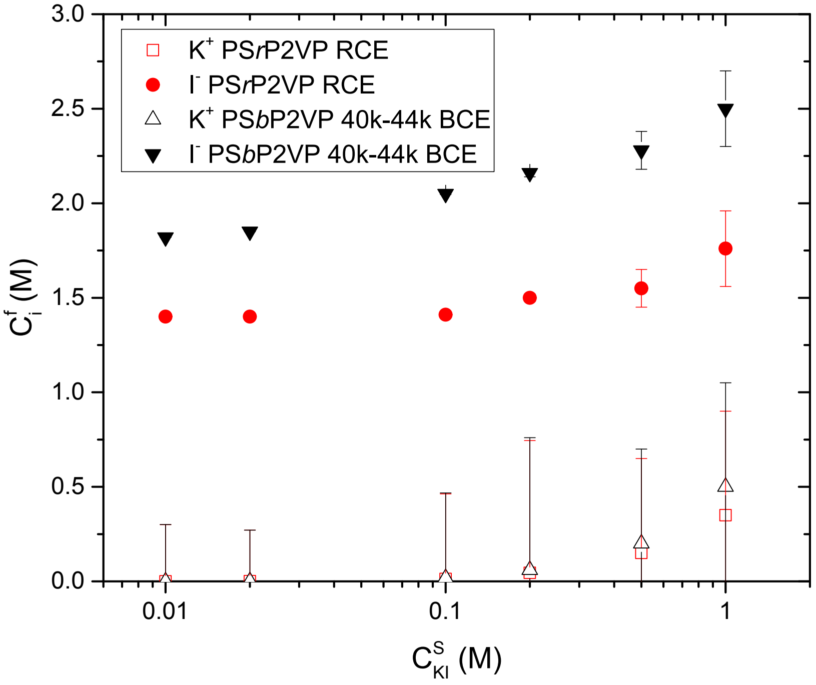

Figure S1. Co-ion and counterion concentration values in the RCE and PS*b*P2VP 40-44k BCE thin films as a function of external KI_{aq} concentration.

Molecular Dynamics

A. Iodide-Water and Pyridinium Radial Distribution Functions

a)

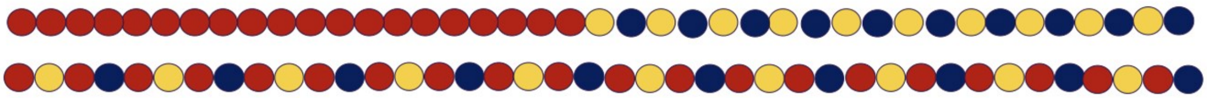

b)

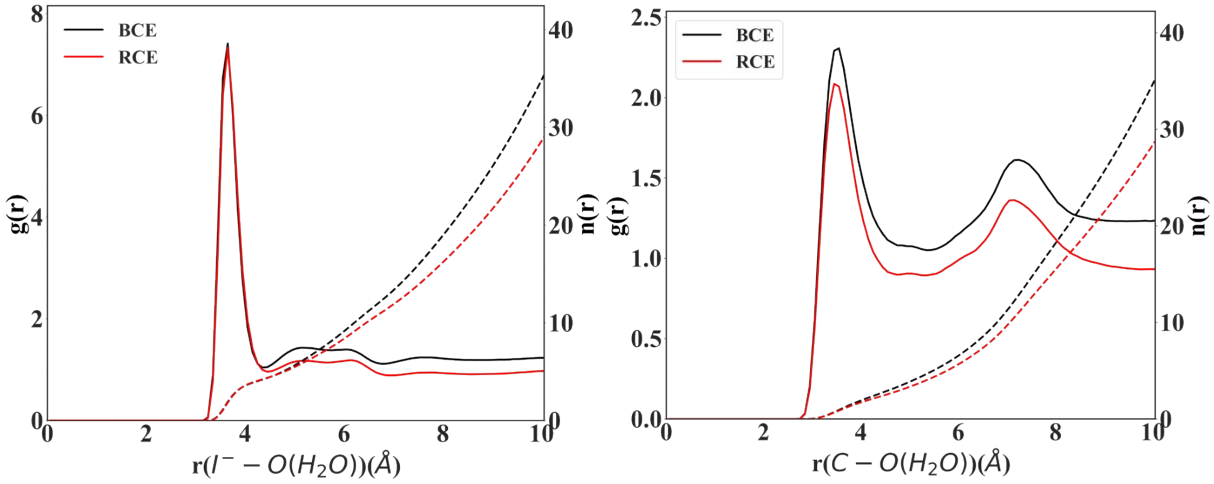

Figure S2. a) A schematic representation of the BCE (top) and RCE (bottom) polymer chains used in the simulation. Red circle stands for styrene, yellow for pyridine and blue for pyridinium. b) The radial distribution function, g(r), (solid lines) and coordination number, n(r), (dashed lines) for I-O (H_2O) (left) and C (C from -CH_3 group attached to N) -O (H_2O) (right) for the BCE and RCE.

The distance dependent coordination number, n(r), is defined as:

$$n(r) = \int_0^{L/2} 4\pi r^2 \rho g(r) dr$$

where g(r) is the ion-water radial distribution function, ρ is the average water density, and L is the length of the simulation box. The value of n(r) at the first minimum of the corresponding ion-water radial distribution function gives the average number of water molecules around the ion in the first solvation shell and it is tabulated for the I^- and pyridinium cation in Table 1.

B. Water Self- Diffusion coefficients

The iodide and water diffusion coefficients were obtained from the slope (diffusion constant is 1/6 of the slope) of the mean square displacement (MSD). The MSD for the iodide and water molecules is plotted in Figure S3.

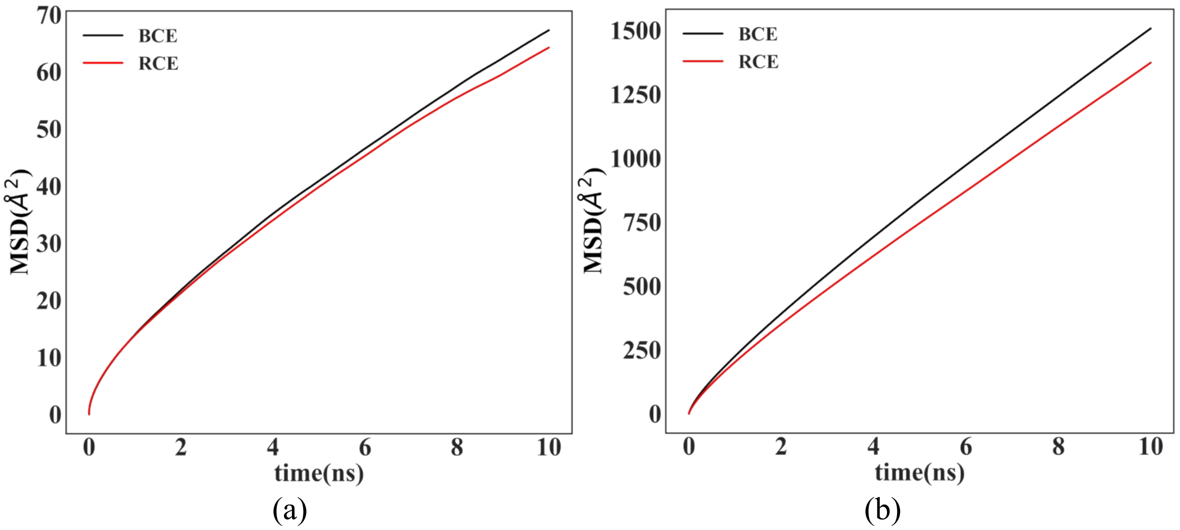

Figure S3. Mean square displacement for (a) iodide (b) water molecules.

C. Orientation Dynamics

The rotational time-correlation function $C(t) = < P_2(\hat{u}(t) \cdot \hat{u}(0)) >$ was calculated where P_2 is the second Legendre polynomial and $\hat{u}$ is the H-H unit vector of a water molecule. The long-time decay is fit to an exponential and the rotational time is the integral of this time correlation function from 0 to infinity.[1] Figure S4 shows the time correlation function for water in the BCE and RCE.

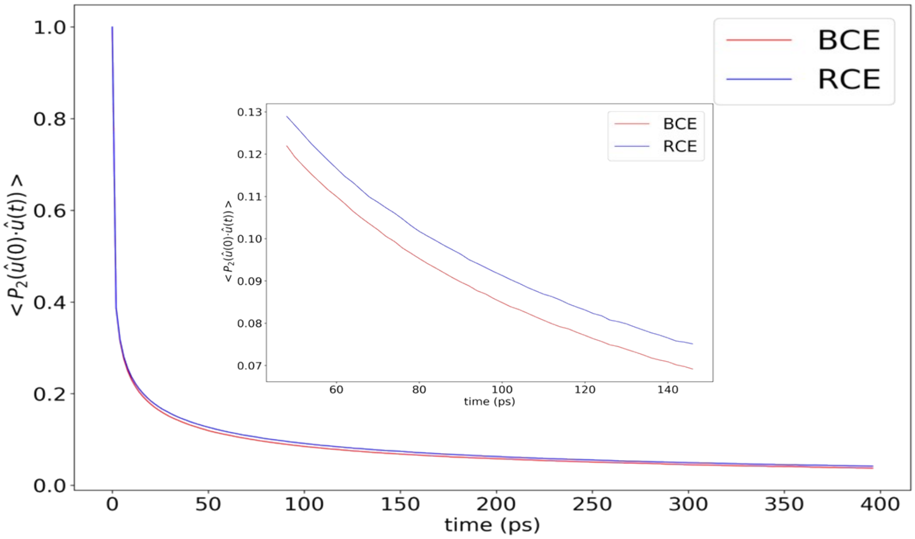

Figure S4. The rotational time correlation function for the waters in the BCE and RCE case. The inset shows the long-time decay.

D. Hopping

In order to determine the iodide hopping rate, the density of pyridinium ions in the solvation shell was identified using the pyridinium methyl C atom ----iodide distance. Any pyridinium ion that is within a distance of the iodide ion that corresponds to the first minimum in the corresponding radial distribution function (see Figure S5) is taken to be part of the iodide ion's solvation shell.

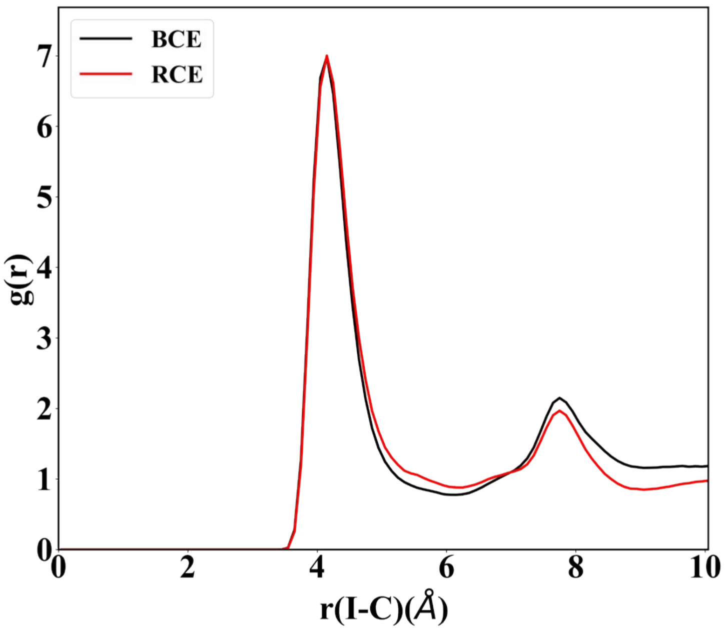

Figure S5. The radial distribution function, g(r) for C (methyl group attached to N atom of pyridinium) and I^- for the BCE and RCE.

References:

1. R. Kumar and J. L. Skinner, *J. Phys. Chem. B,* 2008, **112,** 8311-8318.
